# Macrophage-cancer hybrid membrane-coated nanoparticles for targeting lung metastasis in breast cancer therapy

**DOI:** 10.1186/s12951-020-00649-8

**Published:** 2020-06-16

**Authors:** Chunai Gong, Xiaoyan Yu, Benming You, Yan Wu, Rong Wang, Lu Han, Yujie Wang, Shen Gao, Yongfang Yuan

**Affiliations:** 1 Department of Pharmacy, Shanghai Ninth People’s Hospital, Shanghai JiaoTong University School of Medicine, Shanghai, 201999 China; 2 Department of Pharmaceutics, Changhai Hospital, Second Military Medical University, Shanghai, 200433 China

**Keywords:** Hybrid membrane, Biomimetic nanoparticles, Multi-target capability, Metastasis breast cancer, Chemotherapy

## Abstract

Cell membrane- covered drug-delivery nanoplatforms have been garnering attention because of their enhanced bio-interfacing capabilities that originate from source cells. In this top-down technique, nanoparticles (NPs) are covered by various membrane coatings, including membranes from specialized cells or hybrid membranes that combine the capacities of different types of cell membranes. Here, hybrid membrane-coated doxorubicin (Dox)-loaded poly(lactic-co-glycolic acid) (PLGA) NPs (DPLGA@[RAW-4T1] NPs) were fabricated by fusing membrane components derived from RAW264.7(RAW) and 4T1 cells (4T1). These NPs were used to treat lung metastases originating from breast cancer. This study indicates that the coupling of NPs with a hybrid membrane derived from macrophage and cancer cells has several advantages, such as the tendency to accumulate at sites of inflammation, ability to target specific metastasis, homogenous tumor targeting abilities in vitro, and markedly enhanced multi-target capability in a lung metastasis model in vivo. The DPLGA@[RAW-4T1] NPs exhibited excellent chemotherapeutic potential with approximately 88.9% anti-metastasis efficacy following treatment of breast cancer-derived lung metastases. These NPs were robust and displayed the multi-targeting abilities of hybrid membranes. This study provides a promising biomimetic nanoplatform for effective treatment of breast cancer metastasis.

## Background

Breast cancer remains the leading cause of death from malignant tumors in women and accounts for 30% of new cancer diagnoses in women worldwide [1, 2]. Despite the development of novel adjuvant and neoadjuvant chemotherapeutic drugs, breast cancer exhibits a high metastatic potential. Metastatic breast cancer (MBC) remains largely incurable, with a 5-year survival rate of approximately 20% [3, 4]. Effective therapeutic strategies for targeted treatment of MBC are lacking. Rational combination of therapeutic drugs targeting the tumor-innate properties of MBC and offer control of particle size and drug release from the carrier for targeting metastatic cells is a promising strategy for the development of more efficient, less toxic precision medicines for treating MBC [5, 6].

Recently, nanotechnology has been widely used to improve cancer therapy and the application potential in metastatic cancer [7–10]. Our previous studies [11, 12] demonstrated that disulfide cross-linked nano-delivery systems are useful carriers for delivering genes to tumor sites, via enhanced permeability and retention (EPR) effects. Though the EPR effect nano-delivery system may improve extravasation of nano-chemotherapeutics into large, well-vascularized primary tumors, the small dimension, high dispersion, and poor vascularization limit the accessibility of targeted nano-chemotherapeutics to metastatic tumor sites [13, 14]. Hence, nano-chemotherapeutics that can successfully treat metastatic tumors are urgently needed.

Macrophages significantly influence tissue development, homeostasis, and remodeling [15–17]. These physiological processes define the tumor microenvironment, which ultimately influences cancer progression and metastasis [18–21]. Recruited by inflammatory chemokines to the site of inflammation, macrophages affect the endothelium or pannus of inflammatory vessels by interacting with specific ligands and become “resident” [20, 22]. Recently, macrophage membranes were successfully applied to biomimetic delivery systems development to target tumors or inflammatory sites [23–25]. Moreover, some reports suggest that macrophages are involved in the early stages of diffusion, significantly impacting long-term metastatic development, which occurs during late-stage tumor progression [26–28]. During the metastasis of breast cancer, the cancer cells abnormally express vascular cell adhesion molecule-1 (VCAM-1) and infiltrate leukocyte-rich microenvironments; this is related to recurrence in the lungs. Besides, VCAM-1 primes the metastatic cancer cells to bind to metastasis-associated macrophages via counter-receptor α4-integrins to ensure their survival, thereby leading to the formation of metastatic lesions [29–32].

Our previous studies [33, 34] demonstrated that biomimetic nano-delivery systems mediated by macrophage or functional exosome are useful carriers for delivering chemotherapeutics or genes to tumor sites, via active targeting ability. Recently, cell membrane-based biomimetic NPs have received attention for potential drug delivery applications [35–38]. Cell membrane-based NPs drug delivery systems, while preserving the physicochemical properties of the NP core, functionalize the NPs’ cellular membrane with various functional groups that enable immune evasion and specifically target tumor microenvironment [39, 40]. Using these bioinspired strategies, researchers have successfully endowed the NPs with many desirable features. In recent years, a variety of cells other than red blood cells (RBCs) [41], including cancer cells [42–44], macrophages [31, 45], CAR-T cell membrane [46], and stem cells [47, 48], have been used to obtain membrane materials. Specialized NP platforms have been developed with tailored functionalities by coating the NP core with hybrid membranes, formed by fusing natural membranes from different types of cells. For instance, NPs have been functionalized with hybrid membranes obtained by fusing membrane materials obtained from different cell types such as cancer cells and RBCs [49], RBCs, and platelets [50], and RBCs and artificial membranes [51]. However, NPs functionalized with a hybrid membrane combining the membrane materials of macrophages and cancer cells have not been fabricated.

**Scheme 1:**
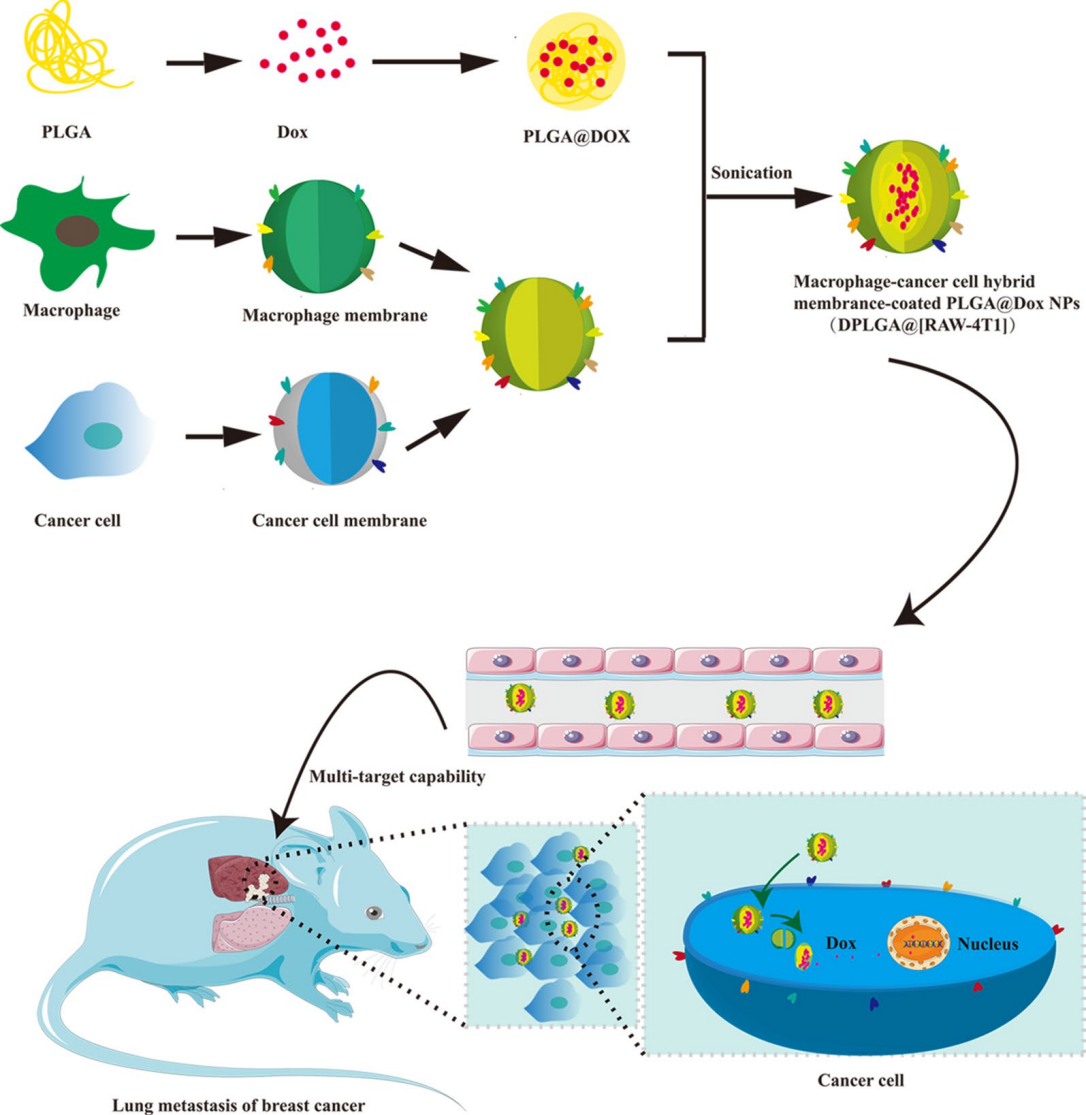
Formation and release of RAW-4T1 hybrid membrane coated doxorubicin (Dox)-loaded PLGA nanoparticles (DPLGA@[RAW-4T1] NPs)

Herein, we report the synthesis of doxorubicin (Dox)-loaded RAW-4T1 hybrid biomimetic membrane camouflaged-poly(lactic-co-glycolic acid (PLGA)) NPs (DPLGA@[RAW-4T1] NPs) for targeting MBCs. The hybrid biomimetic coating, RAW-4T1, obtained from fusing RAW264.7 macrophage membranes (RAW) and 4T1 breast cancer cell membranes(4T1) conferred the NPs with unique functions to improve its anti-metastatic activity (Scheme 1). The multi-targeting capability, biodistribution, and anti-metastatic effect of DPLGA@[RAW-4T1] NPs were systematically evaluated in vitro and in vivo.

## Materials and methods

### Materials

PLGA (50:50, Mw35000, Lack Siomaterials, USA); 4′, 6-diamidino-2-phenylindole (DAPI) (Cayman Chemical, Ann Arbor, MI, USA); 1,1′-dioctadecyl-3,3,3′,3′-tetramethylindotricarbocyanine iodide (DiR), 1,1′-dioctadecyl-3,3,3′,3′-tetramethylindocarbocyanine (DiL) were purchased from Biotium (Fremont, CA, USA); phosphate-buffered saline (PBS), Roswell Park Memorial Institute medium (RPMI-1640), Dulbecco’s modified Eagle’s medium (DMEM), penicillin–streptomycin solution (5 kU mL^−1^), fetal bovine serum (FBS), and trypsin were purchased from Life Technologies (Carlsbad, CA, USA); 1,2-dioleoyl-sn-glycero-3-phosphoethanolamine-*N*-(lissamine rhodamine B sulfonyl), (18:1 Liss RHod PE) and *N*-[6-[(7-nitro-2-2,3-benzoxadiazol-4-yl)amino]hexanoyl]-phytosphingosine, (C6-NBD phytosphingosine) were purchased from Avanti Polar Lipids (Birmingham, AL, USA).

4T1 with stable luciferase expression (4T1-luc cells), 4T1 breast cancer cells, and RAW264.7 macrophage cells were purchased from the Institute of Biochemistry and Cell Biology (Shanghai, China). RAW264.7 cells were cultivated in DMEM media, whereas 4T1-luc and 4T1 cells were cultured in RPMI 1640 media supplemented with 10% FBS. All media contained antibiotics (100 U mL^−1^ penicillin and streptomycin) and 10% FBS.

### Animals

Animal experiments were conducted in accordance with the National Institutes of Health guide for the care and use of laboratory animals (NIH Publication No. 8023, revised 1978) and were approved by the Research Center for Laboratory Animal of The Second Military Medical University of China.

### Preparation of cell membrane fragments

The cell membrane was isolated from RAW264.7 cells or 4T1 cells following a previously reported extrusion approach [52]. Briefly, to extract the cell membrane of RAW264.7, we used a membrane protein extraction kit (Beyotime Biotechnology, Shanghai, China). Both RAW264.7 and 4T1 cells were harvested gently with a rubber scraper, and further separated by centrifugation at 300×*g* for 5 min. The cells were washed with PBS and collected by centrifugation, and then suspended in membrane protein extraction reagent A (adding 1 mM PMSF before use) and cooled down in an ice bath for 15 min. The cells were freeze-thawed three times. The resulting solution was separated by centrifugation at 700×*g* for 10 min at 4 °C. The membrane was obtained by centrifugation at 14,000×*g* for 30 min at 4 °C. Finally, the RAW264.7 or 4T1 cell membranes were frozen, lyophilized, and stored at − 80 °C until analysis. The protein content in the purified cell membrane was determined using the bicinchoninic acid (BCA) protein assay to prepare DPLGA@[RAW-4T1] NPs.

### Membrane fusion study

The process of membrane fusion was observed using the Förster resonance energy transfer (FRET) method [48, 49]. Briefly, the 4T1 cell membrane was stained with DOPE-RhB (detected at an excitation of 560 nm and emission of 583 nm) and C6-NBD (detected at an excitation of 460 nm and emission of 534 nm). The RAW264.7 cell membrane was then added to the DOPE-RhB/C6-NBD (1.74 and 0.17 wt%)-dyed 4T1 cell membrane at different weight ratios (5:1, 4:1, 3:1, 2:1, 1:1, and 0:1), and complete membrane fusion by sonicating at 37 °C for 10 min. The spectrum was recorded from 500 to 650 nm using 470 nm as the excitation wavelength. The fusion process was monitored based on the fluorescence recovery of the donor (C6-NBD).

### Synthesis and characterization of DPLGA@[RAW-4T1] NPs

Briefly, 500 μL Dox (2 mg mL^−1^, prepared and neutralized with triethylamine) was added to a 1 mL solution of PLGA (10 mg mL^−1^ in acetone), and the solution was incubated at 30 ± 2 °C away from light for 2 h with stirring, before precipitating it into water. The organic solvent was removed under vacuum. The RAW264.7 cell membrane, 4T1 cell membrane, or fused RAW-4T1 hybrid membrane was then coated onto the core PLGA NPs by 2 min sonication in a water bath sonicator (Fisher Scientific, Waltham, MA, USA) to form the final cell membrane-camouflaged NPs. To characterize the decoration of the cell membrane, the size and zeta potential of the cell membrane of coated DPLGA@[RAW-4T1] NPs were measured at room temperature after appropriate dilution with distilled deionized water. The particle size and morphology of the cell membrane-coated NPs were investigated by transmission electron microscopy (TEM) (TECNAI G2S-TWIN, FEI, Hillsboro, OR, USA). Furthermore, the Dox release curves from DPLGA@[RAW-4T1] NPs and DPLGA NPs were determined using dialysis tubes containing PBS with different pH values. Briefly, the DPLGA@[RAW-4T1] NPs and DPLGA NPs were placed in the dialysis tubes (MWCO 3.5 kDa) and then soaked in 50 mL of different release media at different pH (pH 7.4, 5.5, and 4.7) containing 0.1% w/v Tween^®^ 20. Different groups of dialysis tubes were placed in a water bath (37 °C) and subsequently stirred at 100 rpm. At predetermined intervals, 200 μL of dialysate were sampled, and the buffer was replaced with 200 μL of fresh supplemented media. The Dox concentration in the solution was detected by measuring the fluorescence with a microplate reader (GloMax-Multi Jr Single Tube Multimode Reader; Promega, Madison, WI, USA). The encapsulation efficiency and the drug loading efficiency were calculated according to the following formulae:

$${\text{Encapsulation efficiency }} = \, \left( {{\text{weight of the loaded drug}}/{\text{weight of the drug in feed}}} \right) \, \times { 1}00\%$$

$${\text{Drug loading efficiency }} = \, \left( {{\text{weight of the loaded drug}}/{\text{total weight of PLGA}}@\left[ {{\text{RAW}} - 4 {\text{T1}}} \right]{\text{ and the loaded drug}}} \right) \, \times { 1}00\%$$

### Protein characterization

To analyze the protein profile of RAW, 4T1, RAW-4T1, and DPLGA@[RAW-4T1] NPs, SDS-PAGE analysis was performed. The surface proteins were derived from RAW-4T1 and hybrid membrane (RAW-4T1) using RIPA buffer (Beyotime, Shanghai, China), followed by staining with Coomassie blue. Specific protein markers were verified by western blot analysis (Na^+^-K^+^-ATPase was used as a reference protein). After transferring the proteins to a nitrocellulose membrane (Thermo Fisher Scientific), the membranes were probed with antibodies against VCAM-1 (66294-1-Ig; Proteintech, Rocky Hill, NJ, USA) and integrin alpha-4 (19676-1-AP; Proteintech). Anti-mouse (Cell Signaling Technology, Danvers, MA, USA; 7076) or anti-rabbit IgG (Cell Signaling Technology, 7074) were conjugated with horseradish peroxidase for signal visualization. We conducted immunogold staining, as reported previously [53]. First, the solution was added to a volume of 4% paraformaldehyde solution, before depositing onto 300-mesh formvar-coated nickel grids and adsorbing to the grids for 20 min and then drying at ambient room temperature (30 ± 2 °C). Next, the grids were soaked for 3 min in 50 mM glycine in PBS and washed twice for 3 min each time. The grid was then transferred to PBS/50 mM glycine for 3 min, which was repeated three times. Next, 5% bovine serum albumin (BSA; Sigma Aldrich, St. Louis, MO, USA) was used to block the grid before incubation with the appropriate diluted first antibody (VCAM-1 or integrin alpha- 4; 1: 20) for 30 min, and then washing with BSA solution six times. Next, the grid was stained with different sizes of colloidal gold that were conjugated to secondary antibodies against mouse or rabbit IgG for 30 min. The grid was washed in PBS/0.5% BSA (blocking buffer) for 3 min, and finally fixed with 1% glutaraldehyde (Sigma Aldrich) for 5 min; the sample was then washed eight times, for 2 min each time, in distilled water. When performing double labeling, this process was repeated with the second label, followed by negative staining with 2% sodium phosphotungstate for 90 s. The samples were observed under an electron microscope (TECNAI G2S-TWIN, FEI) at 80 kV.

### Confocal microscopy

To characterize membrane colocalization, 3,3′-dioctadecyloxacarbocyanine perchlorate (excitation/emission: 488/501 nm; Biotium) was used to stain the membrane of RAW264.7 cells, and 4T1 membrane with 1,1-dioctadecyl-3,3,3,3-tetramethylindodicarbocyanine (excitation/emission: 644/663 nm; eBiosciences, San Diego, CA, USA). DPLGA@RAW NPs, DPLGA@4T1 NPs, or DPLGA@[RAW-4T1] NPs, prepared using these dye-labeled membranes, were analyzed using a fluorescent microscope (Nikon, Tokyo, Japan).

### Optimized conditions for membrane coating

To optimize the coating efficiency of membrane proteins on PLGA NPs, we incubated different amounts of RAW-4T1 with PLGA NPs at various weight ratios (w/w) of the membrane-to-core from 1:4 to 4:1 and then sonicated the NPs for 30 s. We then uncoated RAW-4T1 from the DPLA@[RAW-4T1] NPs by centrifugation for 50 min at 14,500 rpm at 4 °C. The BCA assay was used to determine the concentration of proteins retained on the obtained DPLGA@[RAW-4T1] NPs. To investigate the stability of cell membrane-coated NPs in solution over time, DPLGA NPs, DPLGA@RAW NPs, DPLGA@4T1 NPs, and DPLGA@[RAW-4T1] NPs were resuspended in 1 × PBS buffer, pH 7.4, and the sample sizes were measured by dynamic light scattering (DLS).

### In vitro targeting of DPLGA@[RAW-4T1] NPs

To evaluate the binding ability of cell membrane-coated NPs to 4T1 cells, RAW264.7, G422, RM-1, and 4T1 cells were selected, and flow cytometry analysis was performed. Several types of cells were seeded into 12-well plates, and the cells were cultured until they were 80% confluent. The media was then removed and replaced with fresh medium supplemented with DiL-labeled PLGA@[RAW-4T1] NPs. After 4 h, all groups of cells were collected and suspended in PBS. The uptake of Dox in these groups was measured using a flow cytometer (FACSCalibur; BD Biosciences, Franklin Lakes, NJ, USA).

RAW264.7, G422, RM-1, and 4T1 cells were subcultured at a seeding density of 1 × 10^4^ cells per dish, and cells were further incubated for 12 h. The medium was then removed and replaced with fresh medium supplemented with DiL-labeled PLGA@[RAW-4T1] NPs. DAPI was used to dye the nuclei, which were observed using a confocal microscope after 4 h of incubation.

4T1 cells were subcultured at a seeding density of 1 × 10^4^ per dish, and the cells were further incubated for 24 h. In order to investigate the cellular uptake of membrane-coated nanoparticles, RAW264.7 cell membrane, 4T1 cell membrane, and fused RAW-4T1 hybrid membrane were labeled with PKH67 (a green fluorescent molecular linker used for cell membrane labeling). The medium was then removed and replaced with fresh medium supplemented with Dox, DPLGA NPs, DPLGA@PKH67-4T1 NPs, DPLGA@PKH67-RAW NPs, and DPLGA@PKH67-[RAW-4T1] NPs. DAPI was used to dye the nuclei, which were observed under a confocal microscope after 4 h of incubation.

### Mechanism of the transmembrane

4T1 cells were subcultured in 12-well plates at a seeding density of 2 × 10^5^ per well, and the cells were further incubated for 24 h. To study the transmembrane mechanism of the DPLGA@[RAW-4T1] NPs (1 μg/mL Dox), several specific endocytic inhibitors including chlorpromazine (30 μM, an inhibitor of clathrin),filipin (1 μg mL^−1^, an inhibitor of calveoli), and amiloride (30 μM, an inhibitor of Na^+^/H^+^ exchange) were used to pretreat the 4T1 cells for 1 h, as reported in our previous study [12, 54]. The cells were incubated with DPLGA@[RAW-4T1] NPs. After 2 h, cells were washed twice with PBS, followed by incubation with 400 mL of lysis buffer for 0.5 h. The lysates were centrifuged at 5000 rpm for 3 min and the supernatant was collected. Dox-associated mean fluorescence intensity was analyzed using a fluorescence spectrophotometer at an excitation wavelength of 488 nm and an emission wavelength of 590 nm. The cellular internalization of Dox was also visualized under a confocal laser scanning microscope (Nikon).

### Cytotoxicity and cell apoptosis in vitro

Cell viability was measured by cell-counting kit-8 (CCK-8) assay (Dojindo, Kumamoto, Japan) to assess the cytotoxic effects of DPLGA@[RAW-4T1] NPs on 4T1 cells. Briefly, 4T1 cells were seeded at a density of 8 × 10^3^ per well in 96-well plates, and the cells were further incubated for 24 h; free Dox, DPLGA NPs, DPLGA@4T1 NPs, DPLGA@RAW NPs, DPLGA@[RAW-4T1] NPs were respectively added to each well at concentrations of 0.1 ng mL^−1^ to 10 µg mL^−1^. Next, the media were removed and supplemented with fresh media. The cells were cultivated at 37 °C for 24 and 48 h. Cell viability was measured using the CCK-8 kit, following the manufacturer’s instructions. After adding the CCK-8 solution, the absorbance of each well was detected using a microplate reader (Thermo Fisher Scientific). Each treatment was replicated five times. All data are represented as the mean ± SD. Additionally, cell apoptosis was analyzed by FACS (BD Biosciences). Briefly, after the cells were independently treated with different groups, including free Dox, DPLGA NPs, DPLGA@4T1 NPs, DPLGA@RAW NPs, and DPLGA@[RAW-4T1] NPs (1 μg mL^−1^ of Dox), the cells were subjected to treatment with Annexin V-APC apoptosis Analysis Kit. We detected a minimum of 10,000 cells in each group by FACSCalibur flow cytometry (BD Biosciences). Non-treated cells were used as negative control. The experiment was repeated three times.

### Migration and invasion assays in vitro

A Transwell migration assay was performed with 4T1 cells. The 4T1 cells were treated with or without free Dox, DPLGA NPs, DPLGA@4T1 NPs, DPLGA@RAW NPs, and DPLGA@[RAW-4T1] NPs (1 μg mL^−1^ Dox) resuspended in RPMI-1640/0.1% BSA. Cells at a density of 1 × 10^4^ cells per well (each group was replicated three times) were inoculated into the upper part of 24-well Transwell plates (8 μm pore size). RPMI-1640 supplemented with 20% FBS was added into the lower part of the chamber. After incubation at 37 °C for 24 h, cells remaining on the surface of the upper chamber were wiped off. Cells that had migrated to the lower surface of the chamber were fixed with 4% paraformaldehyde and stained with 0.1% crystal violet solution for 15 min. A microscope (Olympus IX73, Tokyo, Japan) was used to observe the level of migration.

The wound-healing assay was conducted as previously described [55]. The method was as follows: cells at a density of 3 × 10^5^ cells mL^−1^ were inoculated in a culture-insert (Ibidi, Martinsried, Germany) to form a well-demarcated gap. The monolayer cells became gradually confluent after culturing for 24 and 48 h, and then washed 2 times with fresh medium and supplemented with serum-free medium. Free Dox, DPLGA NPs, DPLGA@4T1 NPs, DPLGA@RAW NPs, and DPLGA@[RAW-4T1] NPs (1 μg mL^−1^ Dox) were then added to each well, and the cells were cultured for an additional 2 days. A microscope (Olympus IX73) was used to observe the level of wound healing by acquiring images at 0, 24, and 48 h.

### In vivo distribution of DPLGA@[RAW-4T1] NPs

The establishment of a lung metastatic mouse model was detected by injecting 2 × 10^5^ 4T1-*luc* cells into the tail vein of mice. Prior to the distribution assay, the IVIS Spectrum system (Bio-Real Quick View 3000, Bio-Real Sciences, Austria), bioluminescence imaging (BLI) was conducted 10 min later following intraperitoneal administration of D-luciferin (10 mg mL^−1^, 200 μL) to detect the formation of metastatic lung nodules. The near-infrared dye DiR (1,1′-dioctadecyl-3,3,3′,3′-tetramethylindotricarbocyanine iodide) was used as an imaging probe, which was loaded onto the nanoparticles instead of Dox. Mice were injected with DiR-PLGA@[RAW-4T1] NPs, DiR-PLGA NPs, or free DiR (200 μL, with a DiR payload of 50 μg mL^−1^) (n = 3 for all groups) via the tail vein. The mice were then scanned after 2, 4, and 8 h of administration by IVIS Spectrum system (Bio-Real Quick View 3000; excitation: 745 nm, emission: 800 nm). At 8 h post-injection, the mice were sacrificed, and the organs (including the heart, liver, spleen, lungs, and kidneys) and tumors were separated and washed with saline, and photographs were immediately acquired (with 1 s exposure time). Live Imaging software (Bio-Real Sciences) was used for image analysis.

### In vivo anti-metastasis effect and biosafety

The mouse model of lung MBC was developed as described previously. After 5 days of inoculation, the mice were divided into six groups (n = 5), and the groups were treated as follows by tail vein administration every 3 days: (i) saline (control); (ii) Dox; (iii) DPLGA NPs; (iv) DPLGA@4T1 NPs; (v) DPLGA@RAW NPs; (vi) DPLGA@[RAW-4T1] NPs (a Dox equivalent of 5 mg kg^−1^). All animals were sacrificed on day 15, and the lungs and heart from the animals were carefully isolated. The number of macroscopic metastatic nodules on the lung surface were recorded and photographed. The mouse weights were recorded, and histological analysis of the heart and lung tissues were evaluated by Hematoxylin and eosin (H&E) staining to evaluate cardiotoxicity associated with Dox and to detect metastasis in the lungs, respectively. Moreover, the lifespan of mice in each group was recorded after inoculation.

### Statistical analysis

Data are expressed as the mean ± SD. The mean values between groups were compared by one-way analysis of variance. A value of *p* < 0.05 was regarded as statistically significant.

## Results and discussion

### Characterization of RAW-4T1 hybrid membrane

**Fig. 1:**
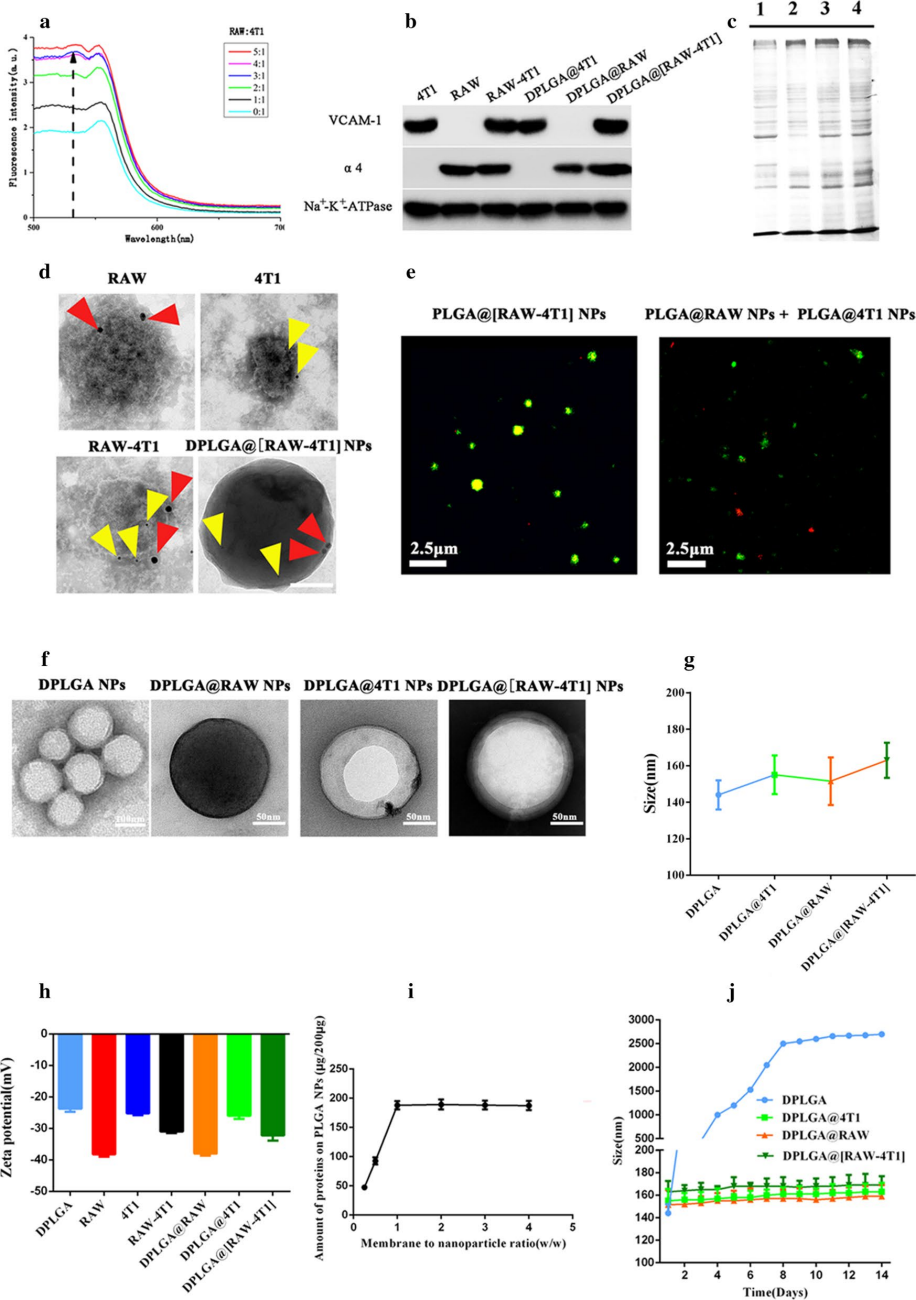
In vitro characterization of hybrid membrane RAW-4T1 and DPLGA@[RAW-4T1] NPs. **a** 4T1 membrane doped with DOPE-RhB and C6-NBD and mixed with an increasing ratio of RAW. The fusion process was monitored as the florescence recovery of the donor (C6-NBD, excitation/emission = 460/534 nm) (4T1: RAW = 4T1 membrane to RAW264.7 membrane protein ratio). **b** Western blot analysis of 4T1, RAW, RAW-4T1 membrane, and DPLGA@[RAW-4T1] NPs for characteristic 4T1 membrane markers VCAM-1, and characteristic RAW264.7 membrane markers α4 (Na^+^-K^+^-ATPase was used as a reference protein). **c** SDS-PAGE analysis of protein retention (1: 4T1, 2: RAW, 3: RAW-4T1 membrane, and 5: DPLGA@[RAW-4T1] NPs). **d** Immunogold TEM images of RAW, 4T1, RAW-4T1, and DPLGA@[RAW-4T1] NPs samples probed for α4 (red arrows, large gold) and VCAM-1 (yellow arrows, small gold), after negative staining with 2% sodium phosphotungstate (scale bar = 50 nm). **e** Images captured by confocal florescent microscopy for the mixture of PLGA@RAW NPs, PLGA@4T1 NPs, and PLGA@[RAW-4T1] NPs (red = 4T1 membrane, green = RAW membrane; scale bar = 5 µm). **f** Representative TEM images of DPLGA NPs, DPLGA@RAW NPs, and DPLGA@4T1 NPs, and DPLGA@[RAW-4T1] NPs negatively stained with vanadium (scale bar = 50 nm). **g** Z-average size of bare DPLGA NPs, DPLGA@4T1 NPs, DPLGA@RAW NPs and DPLGA@[RAW-4T1] NPs were determined by DLS. Data are presented as the mean ± SD (n = 3). **h** Zeta potential of DPLGA NPs, RAW, 4T1, RAW-4T1, DPLGA@RAW NPs, DPLGA@4T1 NPs, and DPLGA@[RAW-4T1] NPs, (n = 3; mean ± SD). **i** Quantification of total proteins on DPLGA@[RAW-4T1] NPs by BCA assay after incubating different amount of RAW-4T1 to the bare PLGA NPs at different membrane-to-polymer weight ratios (w/w). **j** Z-average size of bare DPLGA NPs, DPLGA@4T1 NPs, DPLGA@RAW NPs, and DPLGA@[RAW-4T1] NPs, over 2 weeks in PBS (pH 7.4) (n = 3; mean ± SD)

To verify fusion, the cell membrane of 4T1 cells was stained with the dyes, DOPE-RhB and C6-NBD, which are composed of a pair of FRET probes. FRET interactions were recorded while increasing the weight fraction of RAW. The results are shown in Fig. 1a; as RAW was added, fluorescent signal recovery was recorded at 534 nm. A decrease in the FRET of the two dye-doped 4T1 cell membranes was observed because of the separation of the two membrane materials. The hybrid membranes were prepared at a weight ratio of 1:1 4T1 to RAW and were used to prepare the hybrid DPLGA@[RAW-4T1] NPs. To analyze specific protein markers in the two membrane materials, western blotting analysis was performed (Fig. 1b). Signals for α4 integrins expressed by RAW264.7 cells [31] were observed on RAW membrane, RAW-4T1 hybrid membrane, and DPLGA@[RAW-4T1] NPs. The specific marker, VCAM-1, which is highly expressed in 4T1 cell membranes [56], was found on the 4T1 membrane, RAW-4T1 hybrid membrane, and DPLGA@[RAW-4T1] NPs. Furthermore, SDS-PAGE was performed to observe the protein components in the DPLGA@[RAW-4T1] NPs. As shown in Fig. 1c, the protein markers in the hybrid RAW-4T1 membrane were inherited from the membranes of 4T1 and RAW cells. Additionally, the results of immunogold labeling TEM demonstrated that single DPLGA@[RAW-4T1] NPs simultaneously showed both characteristic markers for 4T1 and RAW (Fig. 1d). To further verify that the hybrid membranes were coated around the surface of the DPLGA NPs, a mixture of DPLGA@RAW NPs and DPLGA@4T1 NPs was prepared using individual fluorescently labeled membranes (Fig. 1e). The results revealed successful fusion of the two membrane materials and confirmed the hybrid membrane coating of the NPs.

### Physicochemical characterization of DPLGA@[RAW-4T1] NPs

TEM observations showed that DPLGA NPs, DPLGA@RAW NPs, DPLGA@4T1 NPs, and DPLGA@[RAW-4T1] NPs (Fig. 1f) displayed a characteristic core–shell like bilayer membrane structures. Consistently, the hydrodynamic size as determined by DLS (Fig. 1g) showed that the original size of DPLGA NPs cores was approximately 144 nm, which was approximately 7–19 nm smaller than those of DPLGA@RAW NPs (151 ± 13.01 nm), DPLGA@4T1 NPs (155 ± 10.6 nm), and DPLGA@[RAW-4T1] NPs (163 ± 9.61 nm). The diameter increases of 10–20 nm were consistent with previous research on the thickness of cell lipid bilayer membranes, which are well-known to be 5–10 nm thick [45, 57]. The zeta potential of the DPLGA NPs changed from − 23.7 to − 31.1 mV, which was similar to the surface charge of pure membrane materials of RAW-4T1 (Fig. 1h). The Dox encapsulation efficiency of the PLGA NPs was 85.4%, and the drug loading efficiency of the PLGA NPs was 9.6%. The BCA assay revealed an optimized membrane-to-polymer ratio of 1:1 (Fig. 1i). Next, DLS was performed to measure the stability of DPLGA@[RAW-4T1] NPs in PBS. As shown in Fig. 1j, the mean diameter of DPLGA@[RAW-4T1] NPs only slightly changed within 14 days in PBS at pH 7.4; this indicates the excellent colloidal stability of the materials, which was attributable to the shielding effect based on the coverage of cell membrane [58].

### Specific targeting 4T1 cell line in vitro

**Fig. 2:**
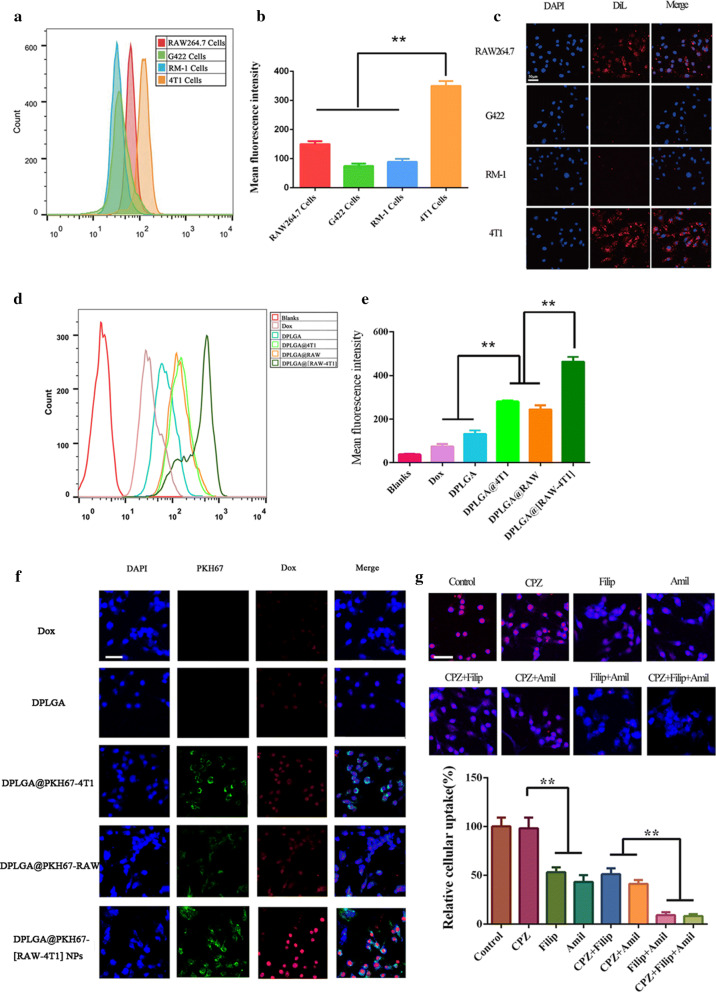
In vitro targeting and transmembrane mechanism of DPLGA@[RAW-4T1] NPs. **a** Flow cytometry detection results and **b** mean fluorescence intensity obtained in four different cell types (RAW264.7, G422, RM-1, 4T1) upon 4 h treatment with DiL-labeled PLGA@[RAW-4T1] NPs. **c** Confocal microscopic images of RAW264.7 cells, G422 cells, RM-1 cells, and 4T1 cells cultured with DiL dyed PLGA@[RAW-4T1] NPs. Scale bar = 50 μm. **d** Mean fluorescence intensity analysis in flow cytometry detection of 4T1 cells incubated with blank solution, free Dox, DPLGA NPs, DPLGA@4T1 NPs, DPLGA@RAW NPs, and DPLGA@[RAW-4T1] NPs. **e** Confocal microscopic observation in 4T1 cells after treatment with blank solution, Dox, DPLGA NPs, DPLGA@4T1 NPs, DPLGA@RAW NPs, or DPLGA@[RAW-4T1] NPs. **f** Cellular internalization of DPLGA@[RAW-4T1] NPs was scanned using a confocal microscope. Scale bar = 50 μm. **g** Impact of inhibitors of endocytic pathways on cell uptake of DPLGA@[RAW-4T1] NPs on 4T1 cells. Data are expressed as the mean ± SD (n = 3). * *p *< 0.05 was regarded to indicate a significant difference between these two groups

We verified the targeting ability of PLGA@[RAW-4T1] NPs to homotypic cancer cells and specificity of the biomimetic interactions. Flow cytometry analysis was conducted to investigate the specificity of DiL-labeled PLGA@[RAW-4T1] NPs to target 4T1 cells. The results demonstrated that the 4T1-RAW membrane coating had an approximately two to fourfold higher average fluorescence intensity in the group of 4T1 cells than in the other groups, demonstrating that DiL-labeled PLGA@[RAW-4T1] NPs specifically targeted 4T1 cells (Fig. 2a, b). Fluorescence microscopy analysis indicated that treating cultured 4T1 cells with DiL-labeled PLGA@[RAW-4T1] NPs in vitro enhanced cellular uptake of NPs as compared to other cell types (Fig. 2c).

To investigate the cellular uptake of DPLGA@RAW NPs, DPLGA@4T1 NPs, and DPLGA@[RAW-4T1] NPs into 4T1 cells, flow cytometric analysis was conducted. The results of flow cytometry indicated higher uptake of DPLGA@[RAW-4T1] NPs, DPLGA@RAW NPs, and DPLGA@4T1 NPs (88.70 ± 2.42%, 72.30 ± 4.32%, and 74.23 ± 1.36%, respectively) than the other nanoparticles (Fig. 2d, e). These results are consistent with previous research showing that macrophages interact with 4T1 cells via α4β1 integrins-VCAM-1 [29, 59]. Compared to DPLGA NPs, DPLGA@RAW NPs and DPLGA@4T1 NPs, the treatment of PLGA@[RAW-4T1]s showed much higher Dox fluorescence (Fig. 2f), indicating that the hybrid membrane RAW-4T1, markedly promoted the internalization in cell of DPLGA NPs because of the retention of homotypic targeting ability from 4T1 membranes and RAW membranes via the α4 integrin-VCAM-1 interactions.

### Internalization mechanism

To evaluate the internalization mechanisms of DPLGA@[RAW-4T1] NPs, chlorpromazine (CPZ), filipin (Filip), and amiloride (Amil) were used to inhibit the clathrin-, caveolin-, and Na^+^/H^+^ exchange-mediated pathways, respectively. As shown in Fig. 2g, preincubation with CPZ did not reduce cellular uptake of DPLGA@[RAW-4T1] NPs. Therefore, endocytosis mediated by clathrin independently contributed little to the uptake of membrane-coated NPs DPLGA@[RAW-4T1] NPs. Filip functions by selectively affecting the formation of caveolae. The cellular uptake of DPLGA@[RAW-4T1] NPs was inhibited by Filip to ~ 53%, indicating that endocytosis mediated by caveolin is a major mechanism in the internalization of DPLGA@[RAW-4T1] NPs in 4T1 cells. Amil inhibits Na^+^/H^+^ exchange, which is involved in s micropinocytosis. Pretreatment with Amil remarkably reduced the cellular uptake of DPLGA@[RAW-4T1] NPs (to approximately 43%), suggesting that the main endocytic mechanism for the uptake of DPLGA@[RAW-4T1] NPs was Na^+^/H^+^ exchange-independent endocytosis. Similarly, to analyze the main pathways contributing to internalization, two endocytosis inhibitors were used for pretreatment with the cells. The same tendency as monotherapy with an inhibitor was observed. Moreover, simultaneous treatment with two or three inhibitors decreased the cellular uptake of DPLGA@[RAW-4T1] NPs to approximately 9% (Fig. 2g), indicating that both the caveolar- and Na^+^/H^+^ exchange-mediated pathways were dominant in the process of cellular uptake, while the pathway mediated by clathrin was not the primary internalization mechanism of DPLGA@[RAW-4T1] NPs in 4T1 cells, which was similar to a previously reported internalization mechanism of extracellular vesicles [60].

### In vitro dox release

**Fig. 3:**
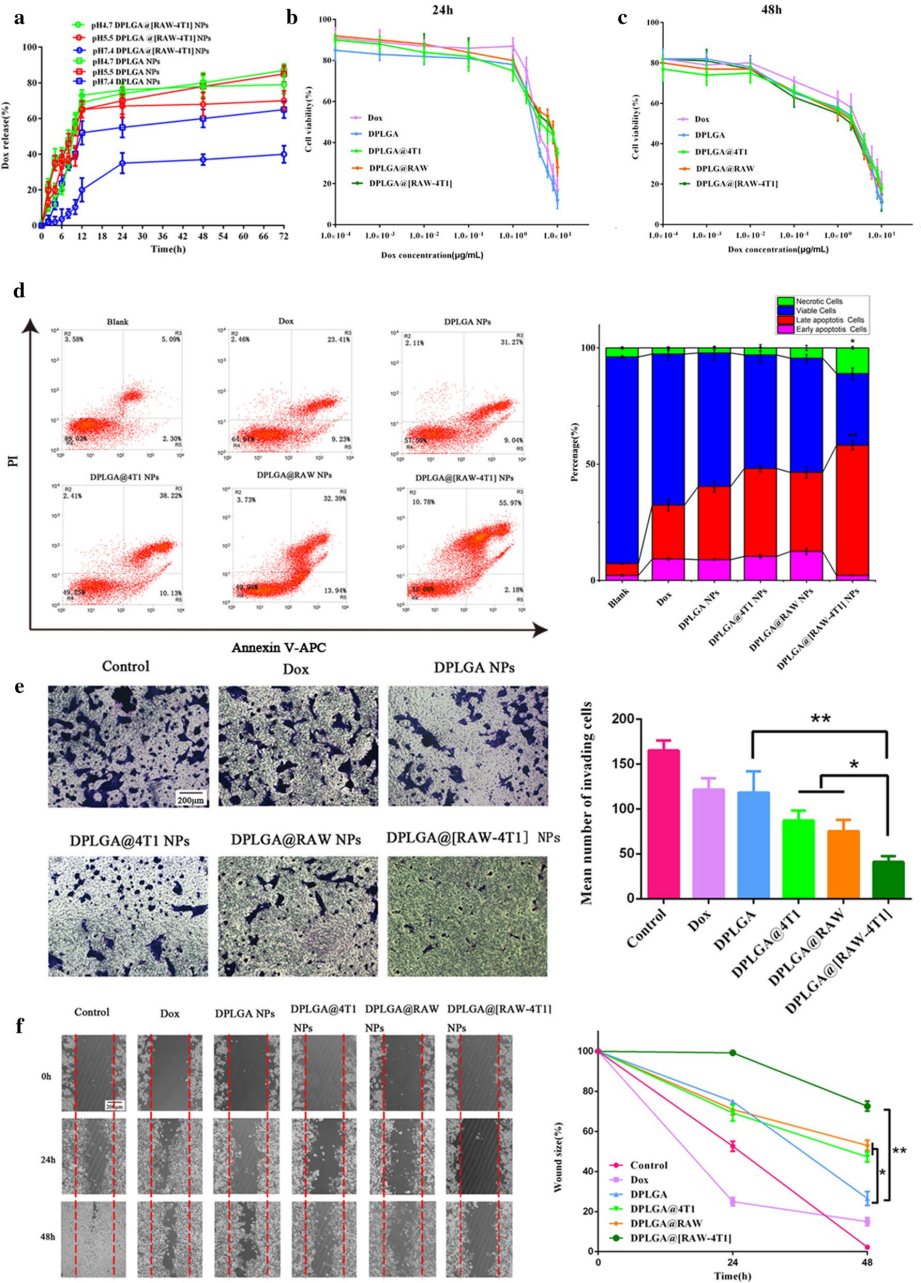
In vitro drug release and therapeutic efficacy of 4T1 cells treated with different formulations. **a** Dox release from DPLGA NPs or DPLGA@[RAW-4T1] NPs at pH 4.7, 5.5, and 7.4. Cell viabilities of 4T1 cells incubated with Dox, DPLGA NPs, DPLGA@4T1 NPs, DPLGA@RAW NPs, and DPLGA@[RAW-4T1] NPs for 24 (**b**) and 48 h (**c**). **d** Analysis of apoptosis in 4T1 cells incubated with different groups for 48 h, followed by detection using the apoptosis kit of Annexin V-APC/PI by flow cytometry. **e** Invasion assay of cells treated with free Dox, DPLGA NPs, DPLGA@4T1 NPs, DPLGA@RAW NPs, and DPLGA@[RAW-4T1] NPs. Scale bar = 200 μm. **f** Scratch assay of cells treated with free Dox, DPLGA NPs, DPLGA@4T1 NPs, DPLGA@RAW NPs, and DPLGA@[RAW-4T1] NPs revealed wound closure. Scale bar = 200 μm. (mean ± SD, n = 3). * *p *< 0.05, ** *p *< 0.01, *** *p *< 0.001

PLGA NPs are considered as ideal drug delivery carriers of antitumor drugs. Lysosomes and endosomes exhibit pH values of 4–5 and 5–6, respectively, which is an effective trigger for intracellular drug release [31, 61, 62]. Analysis of Dox release from the DPLGA@[RAW-4T1] NPs and DPLGA NPs in media with different pH values (pH 4.7, 5.5, and 7.4) showed that Dox release was pH-dependent (Fig. 3a). Over time, the accumulative release percentage increased at lower pH conditions (pH 4.7 and 5.5) and slowly increased at pH 7.4. At pH 7.4, DPLGA@[RAW-4T1] NPs released less than 40% of the loaded Dox within 72 h, after which the release profile reached a plateau; however, the group of DPLGA NPs released 65% of the loaded Dox, indicating that DPLGA@[RAW-4T1] NPs were more stable and had lower Dox leakage than DPLGA NPs in the physiological environment. Cumulative release of DPLGA@[RAW-4T1] NPs at 72 h approached 70% and 79% at pH 5.5 and 4.7, respectively, which was much higher than that at pH 7.4 (approximately 40%). This unexpected result may be due to the increased solubility of Dox under low pH, which can cause Dox to diffuse from the membrane-coated NPs to the surrounding medium [63, 64].

### In vitro therapeutic effect

The cytotoxicity and apoptosis of DPLGA@[RAW-4T1] NPs, DPLGA@RAW NPs, and DPLGA@4T1 NPs in 4T1 cells were examined by cell viability and apoptosis assays. The groups treated with free Dox, DPLGA NPs, DPLGA@4T1 NPs, DPLGA@RAW NPs, and DPLGA@[RAW-4T1] NPs inhibited the proliferation of 4T1 cells in a time- and dose-dependent manner for 24 and 48 h (Fig. 3b, c). The cells treated with DPLGA@4T1 NPs, DPLGA@RAW NPs, and DPLGA@[RAW-4T1] NPs showed higher IC_50_ values than those treated with Dox and DPLGA NPs for 24 h (4.70 μg mL^−1^ for DPLGA@4T1 NPs, 5.13 μg mL^−1^ for DPLGA@RAW NPs, 4.82 μg mL^−1^ for DPLGA@[RAW-4T1] NPs, 3.71 μg mL^−1^ for free Dox, 2.71 μg mL^−1^ for DPLGA NPs); this higher IC_50_ is likely attributable to the uninterrupted release of Dox from DPLGA NPs, DPLGA@4T1 NPs, DPLGA@RAW NPs, or DPLGA@[RAW-4T1] NPs, and diverse uptake mechanisms for free drugs, drug-carried NPs, and membrane-coated NPs [65, 66]. After 48 h of treatment with DPLGA@[RAW-4T1] NPs, the lowest IC_50_ was observed (0.47 μg mL^−1^ for DPLGA@4T1 NPs, 0.44 μg mL^−1^ for DPLGA@RAW NPs, 0.41 μg mL^−1^ for DPLGA@[RAW-4T1] NPs, 0.99 μg mL^−1^ for Dox, 0.54 μg mL^−1^ for DPLGA NPs), which was 2.41-fold lower than that in the Dox group, demonstrating that DPLGA NPs decrease cell viability when delivered by hybrid membranes.

Moreover, flow cytometry analysis was performed to count apoptotic cells (Fig. 3d). At 1 μg mL^−1^ Dox, the percentage of cell apoptosis (early and late apoptosis) induced by DPLGA@[RAW-4T1] NPs was approximately 58.15%, which was much higher than in the group of free Dox and DPLGA NPs (*p *< 0.05). The higher apoptotic potential of DPLGA@[RAW-4T1] NPs compared to that of the free drugs and DPLGA NPs, might be due to better internalization and the sustained release behavior of the hybrid membrane-coated nanoparticle system [67, 68]. Importantly, treatment with DPLGA@[RAW-4T1] NPs resulted in significantly more necrotic cells (*p* < 0.05) and cells in late apoptosis (*p* < 0.01) compared to that observed in other treatments. Extant literature reports that cellular response to the cytotoxic agents (e.g., doxorubicin and cisplatin) includes life cycle inhibition or induction of death by apoptosis. Moreover, excessively high concentrations of such cytotoxic substances can induce cell death by necrosis [69–71]. The hybrid membrane-coated nanoparticle system-based specific internalization is expected to increase the intracellular concentration of drugs that might act synergistically to exhibit enhanced anticancer effects.

### Migration and invasion assays in vitro

Transwell and scratch-wound healing migration assays were conducted to determine whether DPLGA@[RAW-4T1] NPs can directly suppress 4T1 cell migration in vitro. The Transwell and scratch-wound healing migration assays showed consistent results (Fig. 3e, f). The group incubated with DPLGA@[RAW-4T1] NPs showed the minimum number of invasive cells, which was approximately 75% and 65% less than that of the control and DPLGA NPs groups, respectively (Fig. 3e). Wound healing was analyzed at 48 h (Fig. 3f). The group of DPLGA@[RAW-4T1] showed that the wound size remained at approximately 73%, revealing that cell migration was inhibited, whereas the group of DPLGA NPs was 26.6% (*p *< 0.05). These results revealed that NPs, after hybrid membrane coating, restrained the in vitro metastatic behavior of 4T1 cells.

### Biodistribution and metastasis targeting in lung metastasis model in vivo

**Fig. 4:**
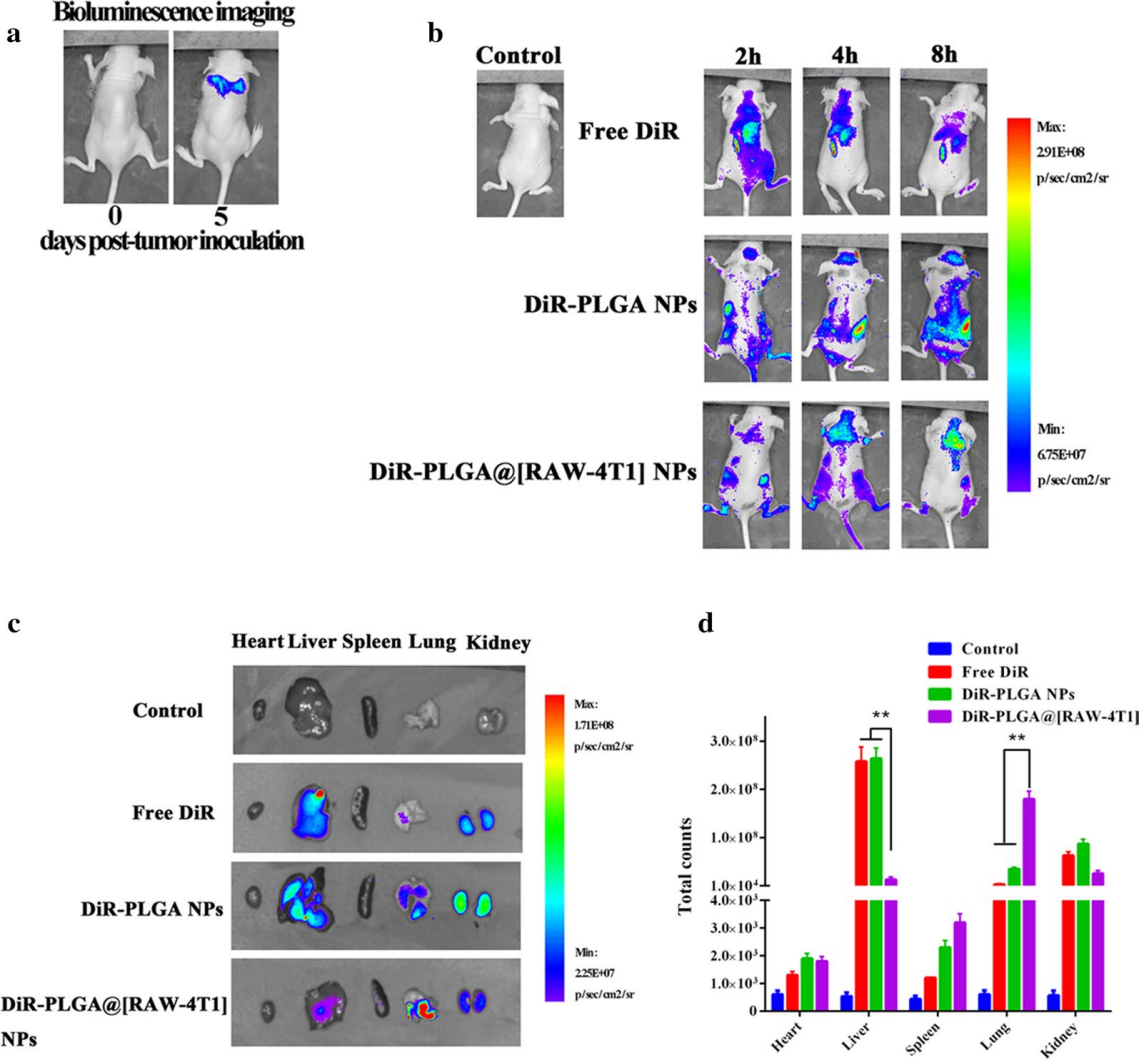
In vivo biodistribution of multiple-targeting DPLGA@[RAW-4T1] NPs after intravenous injection. Typical bioluminescence (BLI) fluorescence images in vivo and ex vivo in model of breast cancer with lung metastasis. **a** BLI images of the same animal showed the progression of 4T1 metastasis in the lungs of mice. **b** Fluorescence images from each group were taken at 2, 4, or 8 h post-injection. **c** Ex vivo fluorescence images of tumor and organs collected from each group were taken 8 h post-injection. **d** Quantification of the ex vivo tumor and organ uptake of DiR and membrane-coated DiR NPs by imaging software (Bio-Real Sciences). Data are expressed as mean ± SD (n = 3)

For these studies, 4T1-luc cells were used to establish a model of lung MBC via caudal vein inoculation [29], which is needed to longitudinally track the metastatic foci of the lung using a bioluminescence assay (IVIS Spectrum system, Bio-Real Quick View 3000, Hercules, CA, USA) prior to the distribution investigation in vivo. Figure 4a displays typical bioluminescence (BLI) photographs of a mouse, indicating the timeline of metastatic progression. Once BLI imaging revealed that the metastatic foci were formed in the lung (day 5), free DiR, DiR-PLGA NPs, and DiR-PLGA@[RAW-4T1] NPs were systemically injected into the animal. The IVIS Spectrum system was used to quantitatively image the fluorescence signals from each group deposited in metastases ex vivo. As shown in Fig. 4b, the majority of DiR accumulated in the liver and kidney, whereas only a weak fluorescence signal was detected in the lungs. The group of DiR-PLGA NPs exhibited a gradually fading fluorescent signal in the liver and lungs 8 h after intravenous injection, as compared to the free DiR group. This difference was attributed to the mononuclear phagocyte system (MPS) and EPR effect because of their small size [72, 73]. Moreover, because of the membrane coating of cancer cells and macrophages, the fluorescent signals were observed in the lung in the DiR-PLGA@[RAW-4T1] NP group, and the signal intensities were gradually enhanced over time. As shown in Fig. 4c, the fluorescent intensity indicated lower accumulation of DiR-PLGA NPs in the lungs. In the liver and kidneys, lower accumulation of DiR-PLGA@[RAW-4T1] NPs was observed in comparison to that of DiR-PLGA NPs. Relying on the hybrid cell membrane coating, DiR-PLGA@[RAW-4T1] NPs showed multi-targeting in lung metastasis model and escaped interception from the liver and kidney. Quantitative analysis (Fig. 4d) indicated that in the DiR-PLGA@[RAW-4T1]-treated group, the signal intensity of DiR was 5.14-fold higher than that in the DiR-PLGA NP group (*p* < 0.01) in the lung tissue, and approximately 20-fold lower than that of free DiR or DiR-PLGA NPs (*p* < 0.01) in the liver. Lesser amount of DiR accumulated in the kidney in the DiR-PLGA@[RAW-4T1] group as compared with that in the free-DiR or DiR-PLGA NP groups (*p* < 0.01). This finding demonstrated that DiR-PLGA@[RAW-4T1] NPs have the multiple-targeting features at the cellular level in vitro as well as targeting characteristics in vivo.

### In vivo anti-metastasis effect and biosafety

**Fig. 5:**
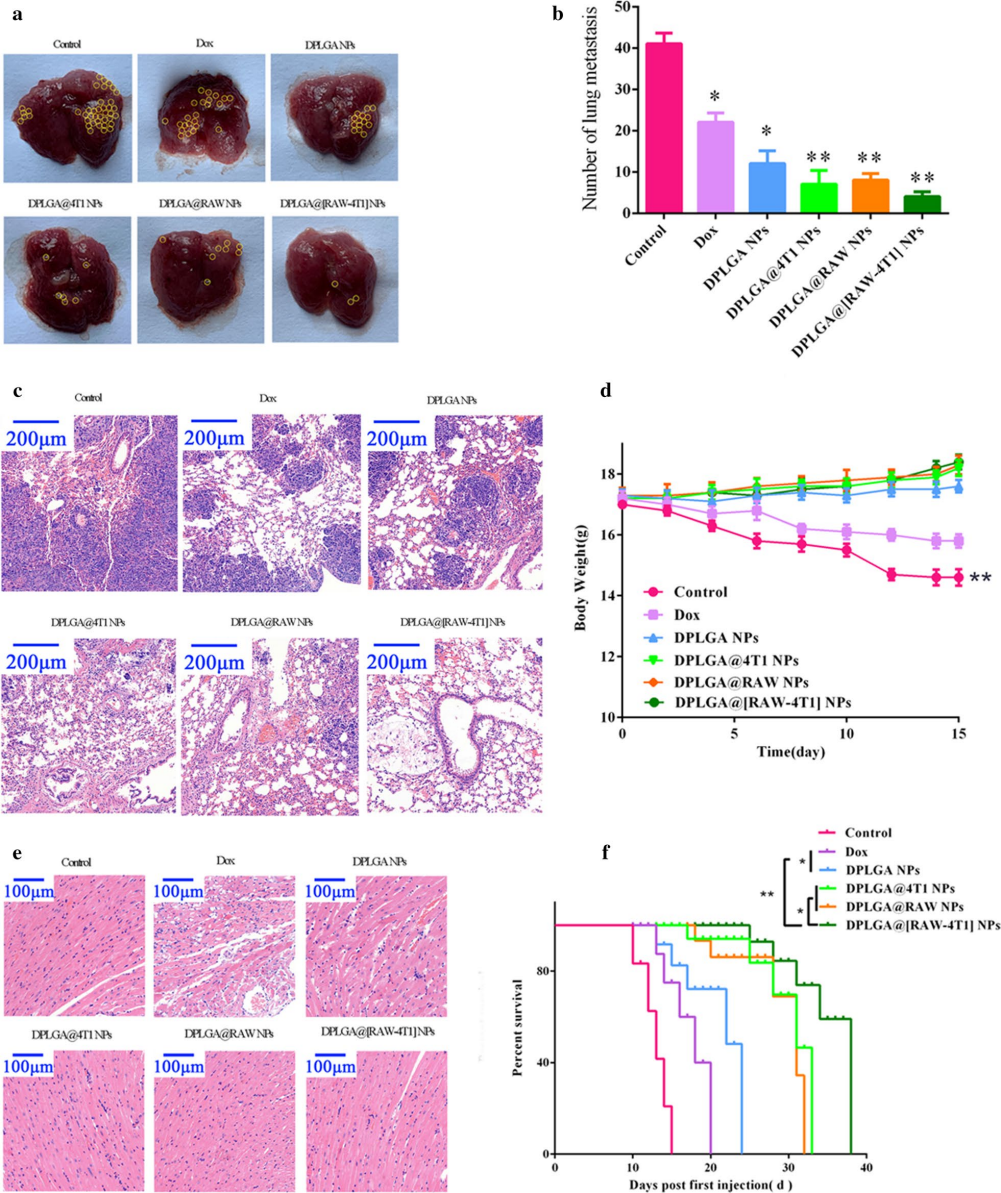
Therapeutic effect of DPLGA@[RAW-4T1] NPs on lung metastatic breast cancer. **a** Representative images of visualized metastatic nodules in the lungs (yellow circles) in each group. **b** Quantitative analysis of pulmonary metastatic nodules in breast cancer in each group. **c** Histological investigation of lung metastasis of breast cancer from each group measured by H&E staining. Scale bar = 200 μm. **d** Weight change in mice during treatment. Data are expressed as the mean ± SD (n = 5). **e** Typical images of H&E staining of heart tissues. Scale bar = 100 μm **f** Mean survival period of mice with 4T1 tumors in different treatment groups. *p < 0.05, **p < 0.01

Finally, the therapeutic efficacy of DPLGA@[RAW-4T1] NPs was verified in the model of breast cancer with lung metastasis. After 4T1 cells were injected, saline (control) Dox, DPLGA NPs, DPLGA@4T1 NPs, DPLGA@RAW NPs, and DPLGA@[RAW-4T1] NPs (Dox equivalent was 5 mg kg^−1^, n = 5) were used to treat mice. At the endpoint, we collected and photographed lung tissues from each group (Fig. 5a), and simultaneously recorded the number of nodules from each lung and assessed anti-metastatic efficacy (Fig. 5b). Inoculated by the caudal vein, the lungs serve as the frequent metastatic foci for 4T1 cells. In comparison to control mice, moderately decreased metastatic nodules of breast cancer in the lung were observed in the DPLGA@4T1 NPs, DPLGA@RAW NPs, and DPLGA@[RAW-4T1] NPs groups (*p *< 0.05). However, relative to the saline group (control), the number of metastatic nodules of breast cancer in the lung were dramatically decreased by 80.6%, 77.8%, and 88.9%, respectively (Fig. 5b) in the DPLGA@4T1 NPs, DPLGA@RAW NPs, and DPLGA@[RAW-4T1] NPs groups. Our analysis showed that metastatic nodules were decreased by 33.3–66.7% in the DPLGA@4T1 NPs, DPLGA@RAW NPs, and DPLGA@[RAW-4T1] NPs groups compared to in the DPLGA NPs groups, which may be attributed to the high dispersion, small size, and low vascularization limit and accessibility of targeted nano-chemotherapeutics to metastatic tumor sites. H&E staining was performed on lungs collected from the mice; the results further confirmed the superior anti-metastatic efficacy of DPLGA@[RAW-4T1] NPs (Fig. 5c). The safety profiles of membrane-coated NPs were analyzed based on the weight changes in mice to indicate systemic toxicity [11]. DPLGA NPs and membrane-coated NPs resulted in low systemic toxicity during treatment, while the weight of mice in the saline and Dox groups was decreased (Fig. 5d), possibly because of metastatic nodules in the lungs or systemic toxicity of Dox, respectively. The results of H&E staining in the groups of free Dox-treated group and DPLGA NPs group revealed the presence of myocardial damage, whereas no significant myocardial injury was detected in the groups of membrane-coated NPs groups(Fig. 5e). Long-term survival was observed post-injection. The results showed that the survival period in the saline group was 15 days (Fig. 5f), whereas it was 38 days in the DPLGA@[RAW-4T1] NPs group, 20 days in the Dox group (*p* < 0.01), and 25 days in the DPLGA NPs group (*p* < 0.01), revealing the marked antitumor effects and prolonged survival in the DPLGA@[RAW-4T1] NPs group.

## Conclusions

We successfully fused the macrophage RAW264.7 membrane with the 4T1 cell membrane and prepared macrophage-cancer hybrid membrane-coated DPLGA@[RAW-4T1] NPs for treating MBC in vivo. The surface of hybrid RAW-4T1 membrane coated NPs contained membrane proteins from RAW264.7 and 4T1 cells. Due to the high α4 and β1 integrin expression, the macrophage membrane coating significantly improved the specific metastasis targeting capability of DPLGA@[RAW-4T1] NPs, and the 4T1 membrane coating enabled the targeting of homologous cancer cells, thereby allowing them to actively reach the cancer sites [41, 74, 75]. These NPs also showed inhibitory effects on cell viability, motility, and invasion.

By virtue of the biocompatibility inherited from the PLGA core, the synthesized DPLGA@[RAW-4T1] NPs were successfully used for facilitated anti-metastatic treatment in breast cancer with lung metastasis that resulted in prolonged survival without overt cardiotoxicity. The hybrid cell membrane disguising technology can confer NPs with additional biological functions. Thus, the hybrid cell membrane-disguised nanoplatform is a promising strategy for specific targeting therapy for tumor metastasis.

## Data Availability

All data generated or analysed during this study are included in this published article.

## Abbreviations

NPs: Nanoparticles
Dox: Doxorubicin
PLGA: Poly(lactic-co-glycolic acid)
MBC: Metastatic breast cancer
EPR: Enhanced permeability and retention
VCAM-1: Vascular cell adhesion molecule-1
RBCs: Red blood cells
DPLGA@[RAW-4T1] NPs: Doxorubicin-loaded RAW-4T1 hybrid biomimetic membrane camouflaged-poly(lactic-co-glycolic acid) nanoparticles
DPLGA@4T1 NPs: Doxorubicin-loaded 4T1 cells membrane camouflaged-poly(lactic-co-glycolic acid) nanoparticles
DPLGA@RAW NPs: Doxorubicin-loaded RAW264.7 cells membrane camouflaged-poly(lactic-co-glycolic acid) nanoparticles
DPLGA NPs: Doxorubicin-loaded poly(lactic-co-glycolic acid) nanoparticles
FRET: Förster resonance energy transfer
TEM: Transmission electron microscopy
BSA: Bovine serum albumin
MPS: Mononuclear phagocyte system
H&E: Hematoxylin and eosin
